# Using genomic selection to correct pedigree errors in kiwiberry breeding

**DOI:** 10.1007/s11032-025-01552-6

**Published:** 2025-03-11

**Authors:** Daniel Mertten, Catherine M. McKenzie, Susan Thomson, John McCallum, Dave Andersen, Samantha Baldwin, Michael Lenhard, Paul M. Datson

**Affiliations:** 1 The New Zealand Institute for Plant and Food Research Limited (PFR), Auckland, 1142 New Zealand; 2 Institute for Biochemistry and Biology, University of Potsdam, 14476 Potsdam-Golm, Germany; 3 The New Zealand Institute for Plant and Food Research Limited, Te Puke, 3182 New Zealand; 4 The New Zealand Institute for Plant and Food Research Limited, Lincoln, 7608 New Zealand; 5 The New Zealand Institute for Plant and Food Research Limited, Motueka, 7198 New Zealand; 6 Kiwifruit Breeding Centre, Auckland, 1142 New Zealand

**Keywords:** Pedigree error, Best linear unbiased prediction, Prediction accuracy, Breeding value, Dioecious

## Abstract

In breeding programmes, accurate estimation of breeding values is crucial for selecting superior genotypes. Traditional methods rely on phenotypic observations and pedigree information to estimate variance components and heritability. However, pedigree errors can significantly affect the accuracy of these estimates, especially in long-lived perennial vines. This study evaluates the effect of pedigree errors on breeding value predictions in kiwiberry breeding and explores the benefits of using genomic selection. We applied Best Linear Unbiased Prediction (BLUP) to estimate breeding values for each genotype for a given trait. Four scenarios with varying degrees of alteration in pedigree-based relationship matrices were used to represent inaccurate relationships between genotypes. Pedigree-based breeding values were compared with genomic estimated breeding values for one vine-related and four fruit-related quantitative traits. The results showed that as the degree of altered population structure increased, the prediction accuracy of pedigree-based breeding values decreased. In contrast, genomic selection, which uses marker inheritance, maintained realised relationships between genotypes, making it a more robust method for predicting genetic merit. In kiwiberries, as in all species of the genus *Actinidia*, only female vines bear fruit. The genotypic merit of fruit-related traits in male genotypes can only be estimated indirectly. Marker-based predictions outperformed pedigree-based predictions, especially for genotypes without phenotypic observations, such as male siblings. This study reviewed the induced population structures and introduced genomic selection into the kiwiberry breeding programme. We demonstrated that genomic selection provides more accurate breeding values by capturing true genetic relationships and reducing the effects of misidentified relationships between individuals.

**Supplementary Information:**

The online version contains supplementary material available at 10.1007/s11032-025-01552-6.

## Introduction

Fruit crop breeding traditionally aimed to improve productivity through increased yields and a wider range of fruit properties utilising relationship information and phenotypic observations among relatives to estimate variance components, heritability, and the merit of a genotype, known as the breeding value. Breeding values of parental genotypes, families, and progeny are very useful for ranking a population and selecting individuals to develop superior lines or for commercialisation. The primary goal of breeding is genetic improvement and the enhancement of heritable traits. Genetic evaluations are often conducted using the best linear unbiased prediction (BLUP) method, particularly for traits with complex inheritance involving many genes with minor effects. Henderson developed the statistical approach of linear mixed models to predict BLUPs, and this method has been widely adopted in animal breeding and increasingly in plant breeding programmes (Henderson 1974; Mrode and Thompson 2014; Muñoz et al. 2014a, b). In animal breeding, genetic value is often estimated indirectly for sex-linked traits using parent–progeny relationships. By extending the estimation to include pedigree information from more relatives, the accuracy of breeding values has improved (Henderson 1975; Lynch and Walsh 1998).

**Fig. 1:**
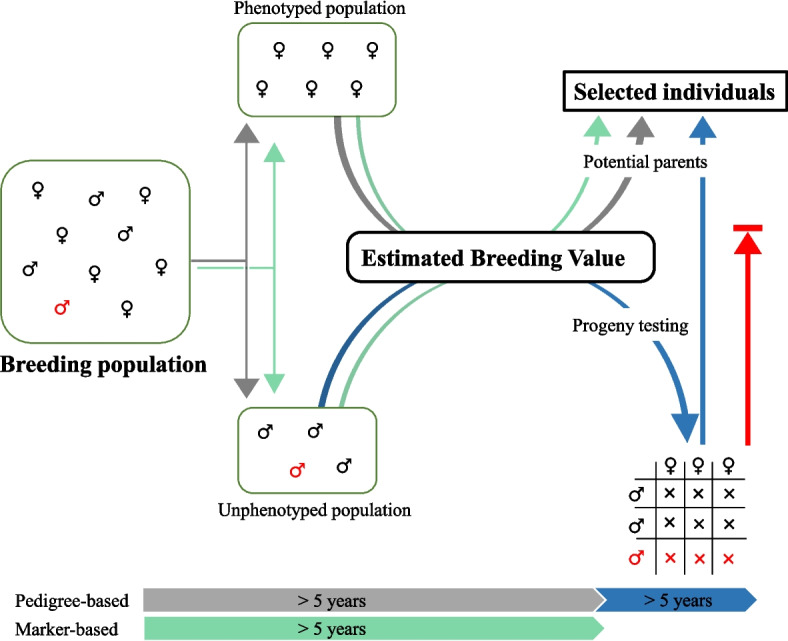
Breeding strategy for parental selection in *Actinidia* species. A breeding population consisting of both female and male genotypes can be divided into phenotyped (female) and unphenotyped (male) groups for sex-linked traits, such as fruit characteristics. By incorporating pedigree-based relationships (‘grey’) among individuals, breeding values for female genotypes can be individually estimated, allowing for the selection of superior genotypes. In contrast, male genotypes require an additional progeny test (‘blue’) to identify superior candidates when using pedigree information. Pedigree-based selection typically requires a minimum of 5 years for female selection and over 10 years for male selection. Errors in pedigree information (‘red’) can lead to incorrect identification of parents. Genomic selection (‘green’) eliminates the need for progeny testing and significantly reduces the time required for the selection process

In plant breeding, a diverse germplasm collection serves as a vital source of genetic variation. This genetic diversity is crucial in breeding programmes as it provides the raw material for selecting and enhancing desirable traits. Founder parental selections originating from natural populations are often misidentified, leading to inaccuracies in perceived relatedness. Another source of misidentified genotypes is contamination during the process of planned crossing (Fig. 1). Next-generation sequencing methods can capture marker inheritance and correct relationship information among individuals (Muñoz et al. 2014b; Moore et al. 2018). Marker inheritance information can help capture population structure and patterns of relatedness in germplasm collections (Navabi et al. 2014; Egan et al. 2019; Koorevaar et al. 2023).

The integration of next-generation sequencing and advanced genetic evaluation methods, which have been successfully applied in traditional fruit crop breeding, can also address the unique challenges posed by the genetic diversity and polyploidy observed in *Actinidia* species, enhancing breeding efficiency and trait selection.

Kiwifruit have been known for centuries but remain one of the more recently commercialised crops. The genus *Actinidia* comprises more than 50 species native to East Asia and Siberia (Ferguson 1991). A new group of *Actinidia* species with commercial potential, known as kiwiberries, such as *Actinidia arguta* (Sieb. and Zucc.) Planch. ex Miq. and *Actinidia melanandra* Franch., is native to East Asia, and produces small fruit with edible skin. While *A. melanandra* is restricted to China, *A. arguta* is also found in Siberia and Japan and both species are highly winter-cold tolerant. Whereas *A. arguta* shows diverse fruit skin colours, ranging from green to purple, *A. melanandra* produces fruit with red to purple flesh and varying skin colours (Williams et al. 2003; Kataoka et al. 2010; Asakura and Hoshino 2016; Zhang et al. 2017). All *Actinidia* species are dioecious, long-lived, climbing perennial vines (Ferguson 1990, 1991; Huang and Ferguson 2007). Two Y-encoded sex determinants linked to sex, Friendly Boy (*FrBy*) and Shy Girl (*SyGl*), have been identified (Akagi et al. 2018, 2019; Akagi and Charlesworth 2019). In sex-heteromorphic crop species, male vines do not produce fruit and therefore require progeny testing to estimate genetic value when only pedigree information is available. However, genomic selection allows for the direct estimation of genetic values for both female and male genotypes, reducing the cost and time required for breeding (Fig. 1) (Cheng et al. 2019).

In addition to the challenges posed by dioecy, the genus *Actinidia* exhibits significant genetic diversity, including varying ploidy levels that influence adaptation (Kataoka et al. 2010; Li et al. 2010; Zhang et al. 2017). Polyploidy resulting from whole-genome duplication plays a significant role in adaptation to environmental conditions (Soltis et al. 2004, 2015; Comai 2005; Baduel et al. 2018). Polyploidy is estimated to account for about 15% of angiosperm speciation events (Wood et al. 2009). The genus *Actinidia* exhibits ploidy levels ranging from diploid (2*x*) to octoploid (8*x*), and higher, with a basic chromosome number of 2*n* = *x* = 29 across all species (Watanabe et al. 1990; Ferguson and Huang 2016). Polyploids are classified based on chromosome set number and inheritance patterns, ranging from autopolyploids to allopolyploids, with a mixed form called allo-autopolyploidy. Autopolyploids arise from genome duplication or the fusion of closely related species, showing non-preferential chromosome pairing during meiosis. Allopolyploids result from the combination of chromosome sets from distantly related species, showing preferential chromosome pairing (Sears 1976; Soltis and Soltis 1999; Soltis et al. 2007). Recent research identified tetraploid *A. arguta* as an autopolyploid resulting from a whole-genome duplication of diploid *A. arguta* (Zhang et al. 2024).

Breeding of autopolyploid crops, such as *A. arguta*, takes much longer owing to the various states of heterozygosity (e.g., Aaaa, AAaa, AAAa), making the elimination of unfavourable alleles more challenging. With advancements in next-generation sequencing and computational capacity, several software programs have been developed to construct relationship matrices considering marker dosage calls for polyploids (Amadeu et al. 2016).

Genomic selection has become a powerful tool in animal breeding and is increasingly adopted in plant breeding, including *A. arguta* (Datson et al. 2017; Hickey et al. 2017; Cheng et al. 2019; Mertten et al. 2023). The increasing availability of next-generation sequencing and genetic markers has revealed pedigree errors in livestock breeding populations. In dairy cattle populations, significant rates of pedigree errors have been estimated (Banos et al. 2001; Wiggans et al. 2012; Pimentel et al. 2024). Especially with large numbers of progeny within crosses, as found in swine, poultry, and crop species, the likelihood of false expected relationships between individuals at certain loci increases. Previous simulation studies of altered pedigrees with different error rates showed a decrease in breeding value prediction accuracy due to misidentified relationships between individuals, leading to incorrect additive variance estimation and breeding value prediction (Long et al. 1990; Muñoz et al. 2014b; Pimentel et al. 2024).

The purpose of this study is to investigate the effect of pedigree errors and validate the beneficial use of genomic selection in kiwiberry breeding. We selected four alterations of population structure representing different degrees of potential pedigree errors to simulate possible crossing errors and the resulting misidentification of parents. For each scenario, the correlation between observed and predicted breeding values was reviewed and compared to the correct relationship information using marker inheritance.

## Material and methods

### Plant population and genotyping

A tetraploid *A. arguta* × *A. arguta* and *A. arguta* × *A. melanandra* seedling population, combined through two incomplete factorial crossing designs, originated from the parental breeding program initiative at The New Zealand Institute for Plant and Food Research Limited (PFR). In 2014, 1832 seedlings resulting from 55 crosses were planted at the PFR Motueka Research Centre. The breeding population comprises two incomplete factorial crossing designs. In the first factorial, two females were crossed with 13 males (2 × 13), while the second factorial involves crosses of 13 females with three males (13 × 3) (Supplementary Table 1). A minimum of 20 seedlings, including both females and males, were planted in groups of seven for each cross. Within the field trial, each cross was represented repeatedly, but no clonal replication was included. Plant spacing within a row was 0.5 m, while the distance between rows was 3.0 m. The seedlings were cultivated on a pergola support system, the prevalent production method in New Zealand. As the plants established, the number of seedlings per cross ranged from a minimum of 2 to a maximum of 80. Six out of the 55 crosses produced fewer than 10 seedlings, while eight crosses yielded more than 40 seedlings. On average, there were 33.3 progeny per cross, with a median of 38 progeny per cross. The plants were grown in the field for two years before fruiting vines were evaluated. Plant maintenance was similar for female and male vines. Two canes from the current growing season were trained horizontally during the summer and retained after winter pruning for vine assessments (Mertten et al. 2023).

Young leaf tissue was collected during spring, and Slipstream Automation (Palmerston North, New Zealand) executed the extraction of DNA. The final dsDNA concentration was standardised to approximately 500 ng per sample. The samples were then vacuum-dried. This was performed to meet the specifications of the high-throughput, targeted multiplex amplicon-sequencing platform Flex-Seq® Ex-L by RAPiD Genomics (Gainesville, FL, USA). RAPiD Genomics designed Flex-Seq probes using a proprietary in-house pipeline. The probes targeted SNPs identified in prior genotyping efforts (Clare et al. 2024). Targets were selected based on heterozygosity and Hardy–Weinberg Equilibrium (HWE), and probes identified as multi-mapping were excluded. A total of 3,300 exonic targets was selected, distributed throughout the gene space. These target generated fragments of 250–350 bp, and paired-end sequencing was performed using the 2 × 150 Illumina NovaSeq platform to provide an expected sequencing coverage of > 100x (Clare et al. 2024). The resulting sequence reads were aligned to the reference genome of *A. chinensis* var. *chinensis* 'Russell' (Tahir et al. 2022) using BWA-MEM (Li 2013) and SAMtools (Danecek et al. 2021) with default parameters. SNP calling was conducted in ANGSD, with region selection based on target intervals (Korneliussen et al. 2014). Dosage estimation for the tetraploid *A. arguta* × *A. arguta* and *A. arguta* × *A. melanandra* population and SNP filtering were carried out using the R-package "Updog" V2. Dosage genotypes were assigned to offspring and parental lines through an empirical Bayesian approach (Gerard et al. 2018). Additional SNP filtering was implemented for quality, allele bias (0.5 < bias < 2), over-dispersion (od < 0.02), and sequencing error (seq < 0.01) (Tahir et al. 2020; Mertten et al. 2023; R Core Team 2023). Genotypes were called under the tetraploid (4*x*) assumption, denoted as 0 (AAAA), 1 (AAAB), 2 (AABB), 3 (ABBB), and 4 (BBBB) (Gerard et al. 2018; Mertten et al. 2023).

### Phenotyping

This study evaluated one vine characteristic (fruit load), and four fruit characteristics (fruit weight, dry matter, ripe soluble solids content, vitamin C content). Fruit load was collected in 2017 and 2018. The assessment involved scoring fruit load on a scale from 0 to 9 (Supplementary Table 2), with vines bearing no fruit receiving a score of 0. Female vines can take more than 3 years to flower due to the extended vegetative stage. As a result, vines without fruit were excluded from this study. Female vines were evaluated based on the number of fruits they developed. Vines were scored based on the number of fruits they carried, with scores ranging from 0.5 for up to four fruits to a maximum of nine for over 500 fruits. Fruit assessments were conducted when fruit maturity was indicated by > 90% black seeds (Beatson et al. 2009). Fruit weight (g) was measured from 2017 to 2019, averaging 30 randomly selected fruits per vine. Dry matter percentage was recorded from 2017 to 2019, with three representative fruits sampled randomly for dry matter percentage calculation (Fenton and Kennedy 1998; Mertten et al. 2023). Ten fruits were randomly subsampled from the harvested fruits and kept at 4 °C for 28 days, followed by 1 day at room temperature to ripen. For ripe soluble solids content (°Brix), three ripe fruits were randomly sampled in 2018 and 2019, and measured using a digital pocket refractometer (ATAGO®) (Beatson et al. 2009; Mertten et al. 2023).

Vitamin C content was quantified utilising a titration method of ascorbic acid content against 2,6-dichlorophenol indophenol (AOAC 1984) and expressed as milligrams of ascorbic acid per 100 g of fresh fruit tissue (mg/100 g fresh weight).

### Exploring the effects of different relationship matrices in linear mixed models

Breeding values were calculated for five quantitative traits using the R-package “ASReml-R” v. 4.1.0.149 (Gilmour et al. 2015; Butler 2021; R Core Team 2023). A univariate linear mixed model equation.

$$y=\mu +Xb+Za+e,$$

Was applied to calculate Best Linear Unbiased Predictors (BLUPs), where $y$ is a one column vector of phenotypic values of each quantitative trait, $\mu$ is the overall population mean, $b$ stands for a vector of fixed effects representing multiple years of observation and the incidence matrix $X$, which connected phenotypic value and fixed effects. The unobserved random effect, related to genotypes is represented by $a$ with $a\sim \text{N}(0,\mathbf{C}{\sigma }_{\text{a}}^{2})$ where ${\sigma }_{\text{a}}^{2}$ is the additive variance and $\mathbf{C}$ is a relationship matrix due the additive effect derived from pedigree (*A*) or marker (*G*).$Z$ is the incidence matrix of genotypes. The variable $e$ represents the random residual effect with $e\sim \text{N}(0,\mathbf{I}{\sigma }_{\text{e}}^{2})$, following a normal distribution with mean of zero and ${\sigma }_{\text{e}}^{2}$, which denotes the residual variance.

ASReml-R employs a restricted maximum-likelihood (REML) methodology, suitable for unbalanced crossing designs (Patterson and Thompson 1971). For each trait, narrow-sense heritability (${h}_{\text{NS}}^{2}$) was calculated on an individual plant basis, taking into account the ratio of the additive variance component to the total phenotypic variance component ${\sigma }_{\text{p}}^{2}$: ${h}_{\text{NS}}^{2}=\frac{{\sigma }_{\text{a}}^{2}}{{\sigma }_{\text{p}}^{2}}$ (Falconer and Mackay 1996).

In this study, different methods of generating a relationship matrix were considered to calculate BLUPs. The R-package “AGHmatrix” v. 2.0.4 was used to generate pedigree-based and marker-based relationship-matrix considering ploidy-level of 4*x* (VanRaden 2008; Kerr et al. 2012; Amadeu et al. 2016; Ashraf et al. 2016; R Core Team 2023).

### Population structure alteration and prediction accuracy

Four pedigree alterations were implemented (Supplementary Fig. 1) and the effect on breeding value prediction and predictive ability was investigated for each alteration. The first alteration (*A*^common^) involved randomly exchanging of female parents within the 2 × 13 factorial, and three male parents of the 13 × 3 factorial for each cross. The second highest degree of pedigree-based relationship change (*A*^distinctive^) was performed considering only male parents (2 × 13 factorial) and female parents (3 × 13 factorial), reflecting the opposite alteration of first model (*A*^common^). The third alteration (*A*^random^) involved a more significant change in pedigree order, achieved through a randomised exchange of female and male parents simultaneously within both factorial crossing designs. This scenario resulted in an increased degree of false relationships between crosses. The fourth randomised modification was made at the progeny level, where the population structure was disrupted by changing the parents of each progeny randomly (*A*^random*^), disrupting full-sib relationship. Pedigree alteration under the different scenarios was performed using the R-package “base” v. 4.3.0 (R Core Team 2023).

### Statistical analysis and visualisation

The summary of trait properties was analysed using the R-package "moments" v. 0.14.1 (Komsta and Novomestky 2022; R Core Team 2023). Five different scenarios of population structures, considering both pedigree-based and marker-based relationship matrices, were analysed and compared. The population structure, influenced by varying degrees of pedigree disruption, was analysed using Principal Components Analysis (PCA). The results were plotted in relation to two common female parents of the 2 × 13 factorial and three common male parents of the 13 × 3 factorial design. This analysis was performed using the “prcomp” function from the R-package “stats” v. 4.3.0 (R Core Team 2023).

A Wald Chi-squared test was performed to evaluate the significance of the fixed effect using the “wald.asreml” function from the ASReml-R package. This test incorporated an identity matrix of genotypes as a random effect and accounted for the independent variable “year of observation”, except for vitamin C content, which had only one year of observation (Gilmour et al. 2015; Butler 2021; R Core Team 2023).

The prediction accuracy of each tested model, considering different relationship matrices and traits, was assessed using the predictive ability, defined as the correlation between the mean observation and the predicted breeding values of female progeny. In this study, the leave-one-out cross-validation (LOO-CV) method was employed to estimate predictive ability. Under LOO-CV, each female was used once to validate the prediction, with their observation masked, while the remaining female progeny constituted the training set. Genomic estimated breeding values were plotted against pedigree-based estimated breeding values for parental and progeny genotypes. Pearson correlation coefficients was calculated using the function “cor.test” from the R-package “stats”. Results were visualised using the R-packages “ggplot2” v. 3.4.2, “patchwork” v. 1.1.1 and “ASRgenomics” v. 1.1.4 (Wickham 2016; Pedersen 2020; Gezan et al. 2022; R Core Team 2023).

## Results

Five quantitative traits were evaluated on a seedling population derived from *A. arguta* × *A. arguta* and *A. arguta* × *A. melanandra* crosses, organised into two factorial designs. The traits assessed included scored fruit load (ranging from 0.5 to 9), average fruit weight (in grams), average dry matter percentage, ripe soluble solids content (in °Brix) and vitamin C content (mg/100 g fresh weight), summarised in Supplementary Table 3. Continuous distributions were observed for all five traits of 1–3 years of replication. Summary statistics for all traits were calculated as follows: fruit load ranged from 0.5 to 9.0, average fruit weight ranged from 1.0 to 17.3 g, average dry matter percentage ranged from 12.0 to 29.3%, ripe soluble solids content ranged from 9.1 to 22.4°Brix, and vitamin C content ranged from 16.8 to 342.5 mg/100 g fresh weight. Normality of the distributions across the population was evaluated, and as expected, all traits exhibited fairly to moderately right-skewed distributions (i.e., positive skewness values) over multiple years. Among all traits, vitamin C content had the highest positive skewness of 0.7 (Supplementary Table 3).

A linear mixed model was used to predict breeding values. The fixed effect of "Year of observation" was included for all traits as a repeated measurement over multiple years. No fixed effects were included for vitamin C content, as it was measured only one season. The Wald Chi-squared Test was used to estimate model improvement and showed a highly significant difference with a *p*-value < 0.001. This suggests a highly significant improvement compared to the linear mixed model without the fixed effect included (Supplementary Table 3).

### Genotyping and population structure

A total of 18,863 unfiltered SNP dosage calls was initially obtained. After applying filtering methods as described by Tahir et al. (2020), 7259 SNPs remained for further analysis.

**Fig. 2:**
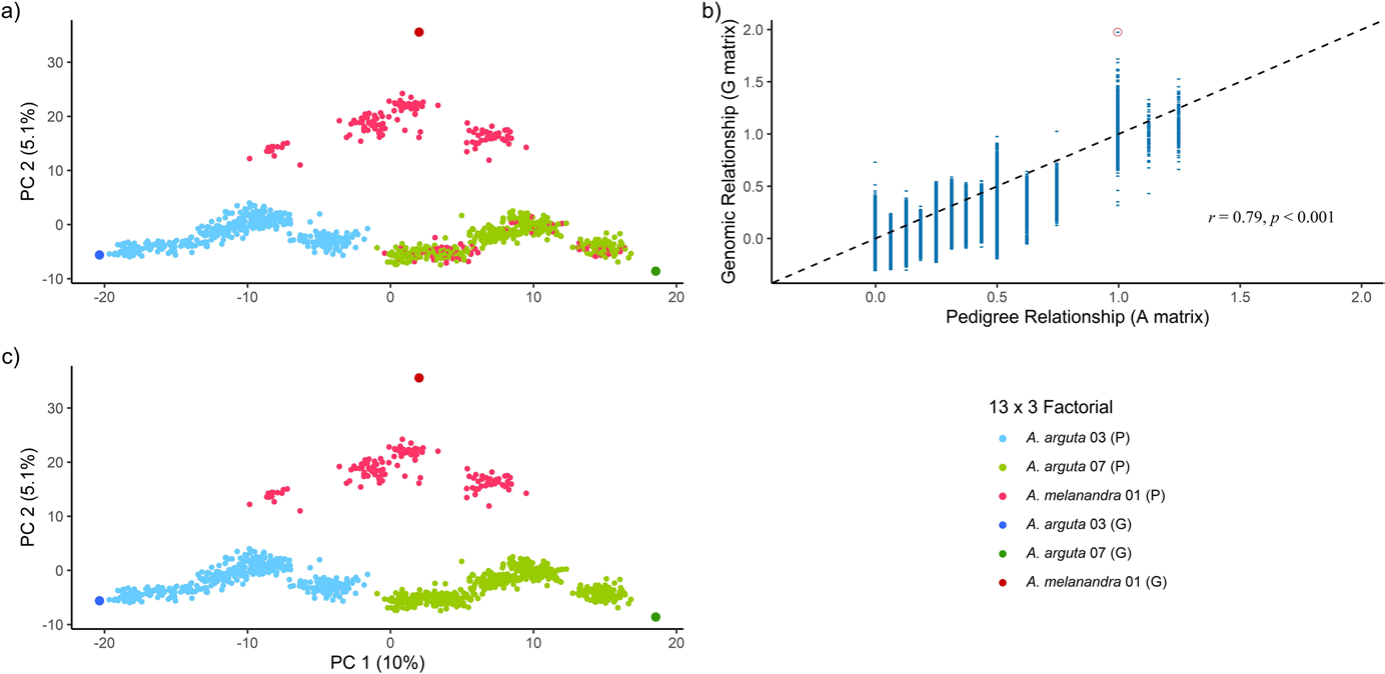
Cross contamination identification using principal component analysis. Parental misidentification in *Actinidia arguta* × *A. melanandra* crosses was identified using PCA (**a**), followed by correction of the male parents (**c**). **b** Correlation between marker-based and pedigree-based relationships after pedigree correction, with distinct genomic relationships across the same A matrix coefficient. The highest discrepancy between G matrix and A matrix was found for *A. melanandra* (red circle) and dashed-line indicate y = x. Colours indicate distinct progeny genotypes (P) related to a particular common parent (G)

We discovered cross-contamination or mislabelled male parent, using marker-based PCA approach (Fig. 2a). The male parent of four crosses within the 13 × 3 factorial design was incorrectly recorded as *A. melanandra* 01 instead of *A. arguta* 07 (Supplementary Table 4). After correcting the pedigree record, based on marker information, the male parent for all crosses was corrected (Fig. 2c). A correlation coefficient of *r* = 0.79 indicated a good estimate of the pedigree-based relationship. However, the largest discrepancy in the diagonal elements between the marker- and pedigree-based relationship matrices was found for *A. melanandra*, with a difference of 1.0 (Fig. 2b).

**Fig. 3:**
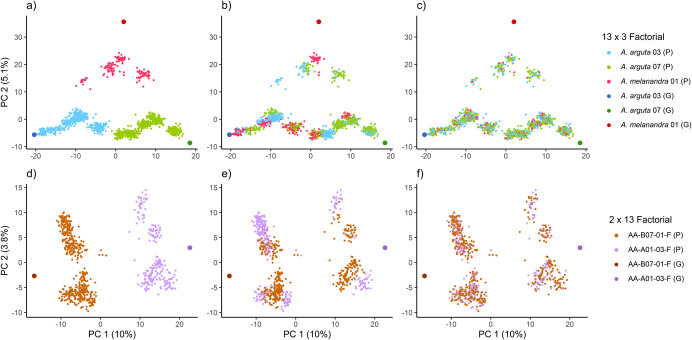
Principal Component Analysis of genetic variation in progeny populations under different pedigree alteration scenarios. **a, d** Unaltered pedigrees (*A*^true^) showing clear distinction of progeny genotypes (P) related to common parental genotype (G). **b**, **e** Randomised common parent scenarios (false male parent in 13 × 3 factorial and false female parent in 2 × 13 factorial) illustrating misclassification (*A*^common^). **c**, **f** Highest degree of alteration with both parents randomised at the level of each progeny (*A*^random*^), significantly disrupting the relationship between individuals through the false parents. The first principal component explains 10% of genetic variation, while the second principal component explains 5.1% of genetic variation between *Actinidia arguta* and *A. melanandra* in the 13 × 3 factorial, and 3.8% in the 2 × 13 factorial. Colours indicate distinct progeny genotypes (P) related to the particular common parent (G)

We further investigated the impact of varying the degree of pedigree alteration in the progeny generation on predicting breeding values. Genotypic dosage calls were utilised in the PCA, and different pedigree data sets were employed to explore various population structures (Fig. 3).

Across all scenarios, a separation of parental genotypes in both incomplete factorials was observed. The first principal component explained 10% of the genetic variation and highlighted a distinction among common *A. arguta* parental genotypes. The second principal component explained 5.1% of the genetic variation between *A. arguta* and *A. melanandra* within the 13 × 3 factorial (Fig. 3a‒c). No further distinction in the 2 × 13 factorial was found when considering the second principal component, which explained 3.8% of the genetic variation (Fig. 3d‒f). When genetic variation within the progeny population was examined using the unaltered pedigree (*A*^true^), a clear distinction of progeny genotypes in relation to the common parent was observed (Fig. 3a and d), as indicated by colour.

The degree of pedigree alteration simulated possible contamination during the crossing procedure and mislabelling during the lifespan of the parental vines. When the common parent was randomly changed for each cross, crosses with a false male parent (13 × 3 factorial) and a false female parent (2 × 13 factorial) showed misclassification by common parents (Fig. 3b and e). The second scenario (*A*^distinctive^), which involved randomising the female parents in the 13 × 3 factorial or the male parents in the 2 × 13 factorial, yielded results equivalent to the previous scenario (*A*^common^), not shown.

A combination of randomising both parental genotypes within the pedigree record (*A*^random^) showed an increasing degree of disrupted population structure through false association of parent for each cross; these results are not visualised. The highest degree of alteration was investigated by randomising both parents at the level of each progeny (*A*^random*^) within each cross (Fig. 3c and f). This approach significantly disrupted the relationship between individuals through their parents.

### Model prediction and breeding value estimation

Breeding values and genetic variance components were calculated using the linear mixed model approach. We explored prediction accuracy by examining the correlation between predicted breeding values (predictive ability) of female progeny under different degrees of pedigree information alteration (Table 1).

**Table 1:** Genetic parameters of assessed quantitative traits: Fruit load (0.5‒9), fruit weight (g), dry matter percentage (%), ripe soluble solids content (°Brix) and vitamin C content (mg/100 g fresh weight). The genetic parameters of additive genetic variance (${{\varvec{\sigma}}}_{{\varvec{a}}}^{2}$), residual variance (${{\varvec{\sigma}}}_{{\varvec{e}}}^{2}$) and narrow-sense heritability (${{\varvec{h}}}_{\mathbf{N}\mathbf{S}}^{2}$) were evaluated using either pedigree-based or marker-based relationship matrix. Predictive ability (PA) was estimated using Leave-One-Out Cross-Validation (LOO-CV) considering different degrees of pedigree alteration and marker-based relationship matrix

Trait	A-Matrix													G-Matrix				
	*A*^true^					*A*^common^		*A*^distinctive^		*A*^random^		*A*^random*^		${\sigma }_{a}^{2}$	${\sigma }_{p}^{2}$	${h}_{\text{NS}}^{2}$	PA	*p*-value
	${\sigma }_{a}^{2}$	${\sigma }_{e}^{2}$	${h}_{\text{NS}}^{2}$	PA	*p*-value	PA	*p*-value	PA	*p*-value	PA	*p*-value	PA	*p*-value					
Fruit Load	2.19	1.73	0.56	0.59	< 0.001	0.29	< 0.001	0.54	< 0.001	0.38	< 0.001	−0.04	0.270	1.44	2.08	0.41	0.54	< 0.001
Fruit Weight	4.95	1.53	0.76	0.54	< 0.001	0.52	< 0.001	0.52	< 0.001	0.53	< 0.001	0.42	< 0.001	5.39	1.64	0.77	0.49	< 0.001
Dry Matter	4.53	2.91	0.61	0.43	< 0.001	0.32	< 0.001	0.38	< 0.001	0.34	< 0.001	−0.04	0.210	3.55	3.23	0.52	0.42	< 0.001
Ripe Soluble Solids Content	3.40	2.42	0.58	0.40	< 0.001	0.25	< 0.001	0.36	< 0.001	0.33	< 0.001	−0.01	0.850	2.09	2.86	0.42	0.41	< 0.001
Vitamin C	3651.32	661.68	0.85	0.57	< 0.001	0.39	< 0.001	0.55	< 0.001	0.43	< 0.001	0.10	< 0.01	1474.73	1804.8	0.45	0.52	< 0.001

Using the model with true pedigree (*A*^true^), we observed medium to high narrow-sense heritability. The lowest heritability was 0.56 for fruit load, while the highest was 0.85 for vitamin C. In the marker-based model, narrow-sense heritability ranged from 0.41 for fruit load to 0.77 for fruit weight (Table 1).

The predictive ability (PA) for both the pedigree-based model (*A*^true^) and the marker-based model ranged from 0.40 to 0.59, showing similar performance. The degree of altered pedigree information affected predictive ability differently. A slight reduction in predictive ability was observed when altering distinctive parents (*A*^distinctive^) and randomising parental order at the cross level (*A*^random^). Changing the relationship through the common parents (*A*^common^) resulted in a more significant reduction of predictive ability. The most substantial reduction was observed when both parents were randomised altered for each progeny (*A*^random*^), with the predictive ability nearing zero. Notably, the predictive ability for average fruit weight was less affected, ranging from 0.42 to 0.54 across all test pedigree-based models (Table 1).

**Fig. 4:**
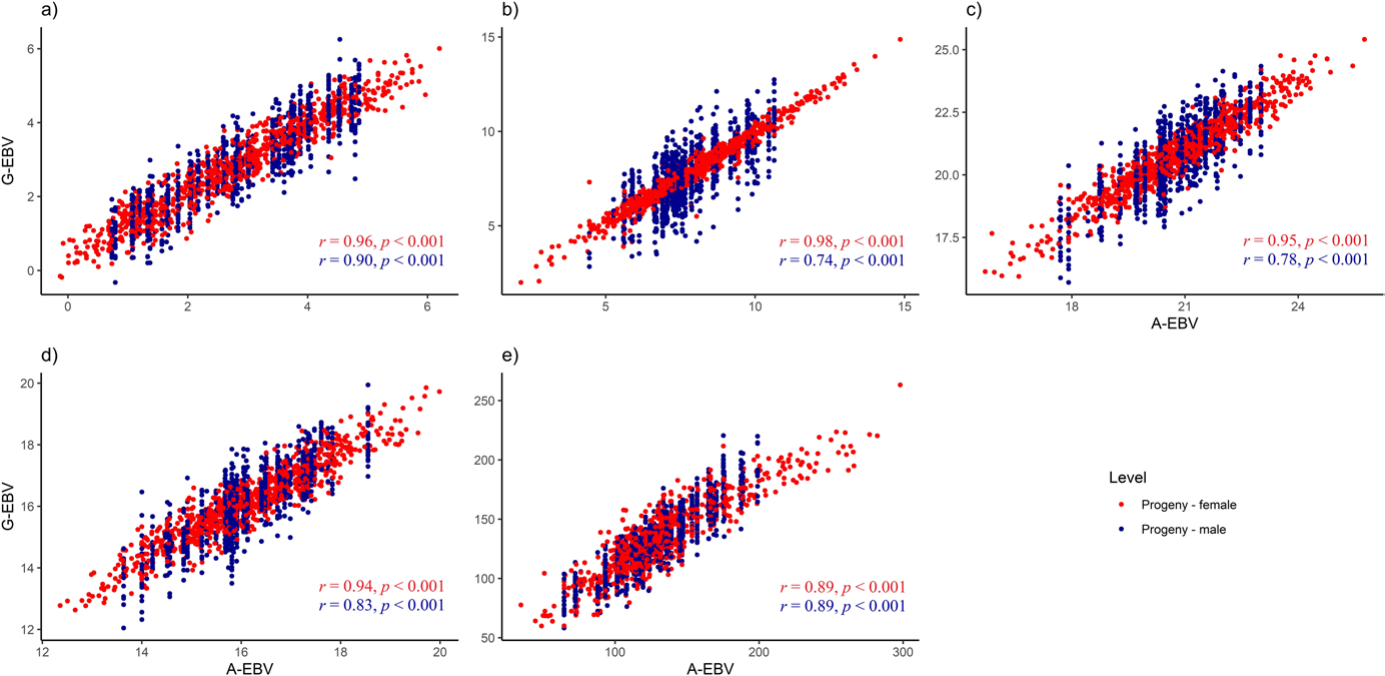
Progeny breeding value correlation. The correlation of pedigree-based (A) and marker-based (G) estimated breeding values (EBV) for progeny genotypes was analysed using five quantitative traits: **a** scored fruit load (0.5‒9), **b** average fruit weight (g), **c** average dry matter percentage (%), **d** ripe soluble solids content (°Brix), and **e** vitamin C content (mg/100 g fresh weight). Progeny genotypes were grouped by sex (female = red, male = blue), with female progeny used as the training set to estimated EBV for male progeny

We also examined the correlation between pedigree-based model predictions and marker-based predictions, assuming the genomic estimated relationship between genotypes as the correct model. A high correlation was observed between pedigree-based (*A*^true^) and marker-based breeding values (Supplementary Fig. 2). Within the progeny generation, the correlation of marker-based predicted breeding values was high for the female progeny used to train the model. In contrast, when only pedigree information was used for male progeny, all individuals within a cross received the same family mean, visible as grouped predictions along the x-axis. Using the marker-based relationship matrix, each male progeny genotype received a unique predicted breeding value (Fig. 4). Across all traits, the correlation coefficient between pedigree-based and marker-based estimate breeding values was in general lower for male progeny (Supplementary Table 5).

Consistent with the effect of pedigree alteration on model prediction ability, we observed a reduction in the correlation between pedigree-based and marker-based breeding value predictions across parental and progeny generations. The strongest correlation was between true pedigree-based and marker-based predicted breeding values for all traits and across generations. The weakest correlation occurred when parental genotypes were randomised for each progeny genotype. Minor differences between female (validation set) and male progeny were observed. Notably, the correlation for average fruit weight was less affected compared to other traits (Supplementary Table 5).

## Discussion

In this study, we investigated the effect of pedigree errors on model prediction ability and breeding value estimation, using a pedigree method compared to genomic selection. Four different scenarios of altered pedigree-based relationship matrices and one marker-based relationship matrix were used to predict breeding values for five quantitative traits: fruit load, average fruit weight, average dry matter, ripe soluble solids content, and vitamin C.

Breeding value prediction relies on the relationship between individuals, which is often probabilistic. Studies in livestock breeding have shown the limitations of using populations distantly related to the reference population with observation records. Unrelated individuals do not contribute to breeding value prediction, or if no performance data are available (Clark et al. 2012).

In crop breeding, the core collection, or "germplasm", is usually selected for desirable traits and based on geographic origin, often with unknown relations between selections. As breeding progresses, the germplasm expands through genotype selection based on phenotypic traits, and relationships between selections become more important (Igartua et al. 1998; Hancock et al. 2002; Koorevaar et al. 2023). Until recently, pedigrees have been used in kiwiberry breeding, to evaluate genetic gain by identifying and crossing parental genotypes. Errors in pedigree records can occur during the process of crossing, seed collection, seedling selection, and maintaining the identity of long-lived perennial vines. In animal breeding such as dairy cattle, it is estimated that there is an error rate of around 10% in pedigrees (Banos et al. 2001; Wiggans et al. 2012; Pimentel et al. 2024). Due to pedigree errors, the assumption of relatedness between individuals is incorrect, leading to inaccurate breeding value predictions (Christensen et al. 1982). We simulated these errors by altering the pedigree. When common or distinctive parents were randomly changed, the relationship between families shifted from half-sib relatedness to potentially false or unrelated, as the pedigree was randomised (Fig. 3b and e). A simulation study in cattle breeding showed that increasing pedigree error rates decrease the correlation between estimated and true breeding values (Pimentel et al. 2024). Our results led to similar conclusions. As the population structure altered at different degrees, the correlation between pedigree-based and marker-based breeding value predictions decreased (Supplementary Table 5). This was consistent for parental breeding value prediction as well as for male and female progeny with masked observations. The greatest decrease in breeding value correlation occurred when both parents were simultaneously altered for each cross and individual (Fig. 3c and f). These errors simulated the misidentification of crosses and incorrect relationships between families and progeny within each family, disrupting the relationship between the training population and genotypes without observations, leading to false breeding value predictions. A minor change in breeding value correlation within the training set was observed, indicating that pedigree errors between genotypes with observations are less affected compared to genotypes without observations, as the direct performance data can offset the inaccuracies caused by pedigree errors.

When relationships between individuals are derived from marker information, individuals can be identical-by-state even when unrelated. Marker-based relationship matrices include information of shared markers, allowing breeding value prediction between probabilistic unrelated individuals. The correlation between pedigree-based and marker-based breeding value predictions suggests that the underlying genetic architecture of traits is consistent for both methods. Parental breeding values benefit from both methods, giving breeders confidence in using either approach (Supplementary Fig. 2). Using pedigree information, individuals within a cross with missing observation records, such as male individuals that do not bear fruit, can only be predicted as a mean. All male individuals within a cross receive the same predicted family-mean and require further progeny testing, which is time-consuming and costly (Cheng et al. 2019). Genomic-based relationships, however, distinguish individuals within families (full-sibs) by marker inheritance (Fig. 4). Based on marker inheritance, breeding values can be estimated for individuals without phenotypic information (Mertten et al. 2023).

In cases of pedigree errors, studies have shown that using the realised relationship matrix can correct these errors, making the prediction of the genetic merit of potential parents more robust (Muñoz et al. 2014b; Gemenet et al. 2019). Our study revealed the necessity of using marker information to identify and correct pedigree records. Especially, when no observations are available, such as male genotypes in kiwifruit, marker information becomes beneficial in selecting potential parents. Further investigation into the effect of interspecific hybrids on genomic selection is needed.

## Supplementary Information

Below is the link to the electronic supplementary material.

Supplementary file1 (DOCX 288 KB)

Supplementary file2 (DOCX 31.9 KB)

## Data Availability

The datasets generated during and/or analysed during the current study are available from the corresponding author on reasonable request.
