# Supplementary material for: Using genomic selection to correct pedigree errors in kiwiberry breeding
Source: Mol Breed. 2025 Mar 11;45(3):33. doi: 10.1007/s11032-025-01552-6 (PMC11896956; doi:10.1007/s11032-025-01552-6)
Supplement: Supplementary file 1 — Supplementary file1 (DOCX 288 KB) [file 11032_2025_1552_MOESM1_ESM.docx]

**Using genomic selection to correct pedigree errors in kiwiberry breeding**

Molecular Breeding

Daniel Mertten*, Catherine M. McKenzie, Susan Thomson, John McCallum, Dave Andersen, Samantha Baldwin, Michael Lenhard, Paul M. Datson

***Corresponding author:**

Daniel Mertten

The New Zealand Institute for Plant and Food Research Limited (PFR)

Auckland 1142, New Zealand

Email: merttendan@gmail.com


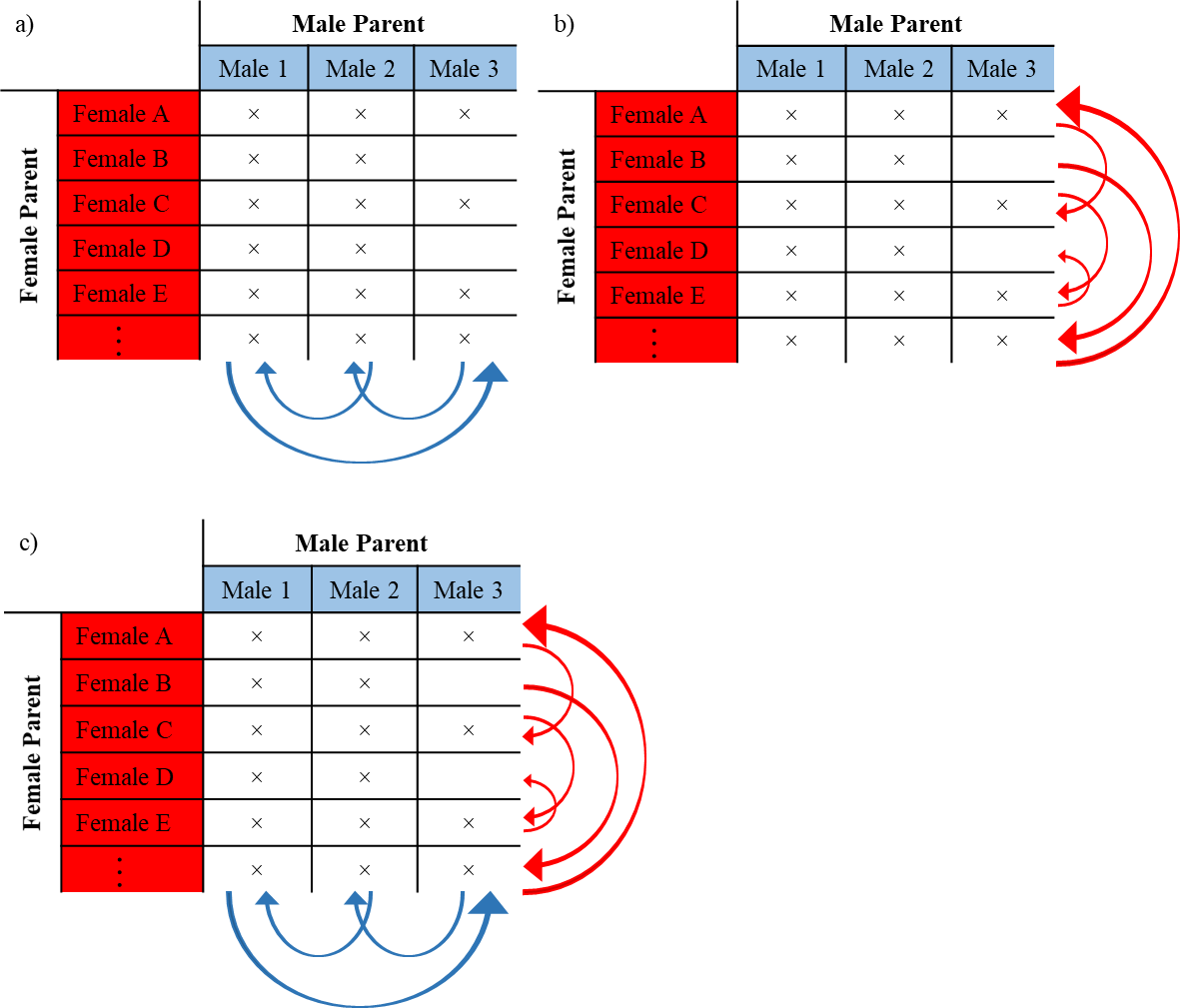


**Supplementary Fig. 1** Illustration of Pedigree Alteration in a 13×3 Factorial Crossing Design. The experimental design is a subset of the 13×3 factorial design, featuring five female parents (A‒E) and three male parents (1‒3). **a)** The model *A*^common^ represents the randomised interchange of male parents within the 13×3 factorial design (blue arrow) and female parents within the 2×13 factorial design for each cross. **b)** In the model *A*^distinctive^, parents with the fewest crosses, specifically female parents in the 13×3 factorial design (red arrow) and male parents in the 2×13 factorial design, were randomly interchanged for each cross. **c)** In the model *A*^random^, both parents for each cross were randomly altered within both factorial designs. The equivalent of model *A*^random*^ is the random alteration of both parents within a cross, effectively breaking the relationship between full siblings (not shown)


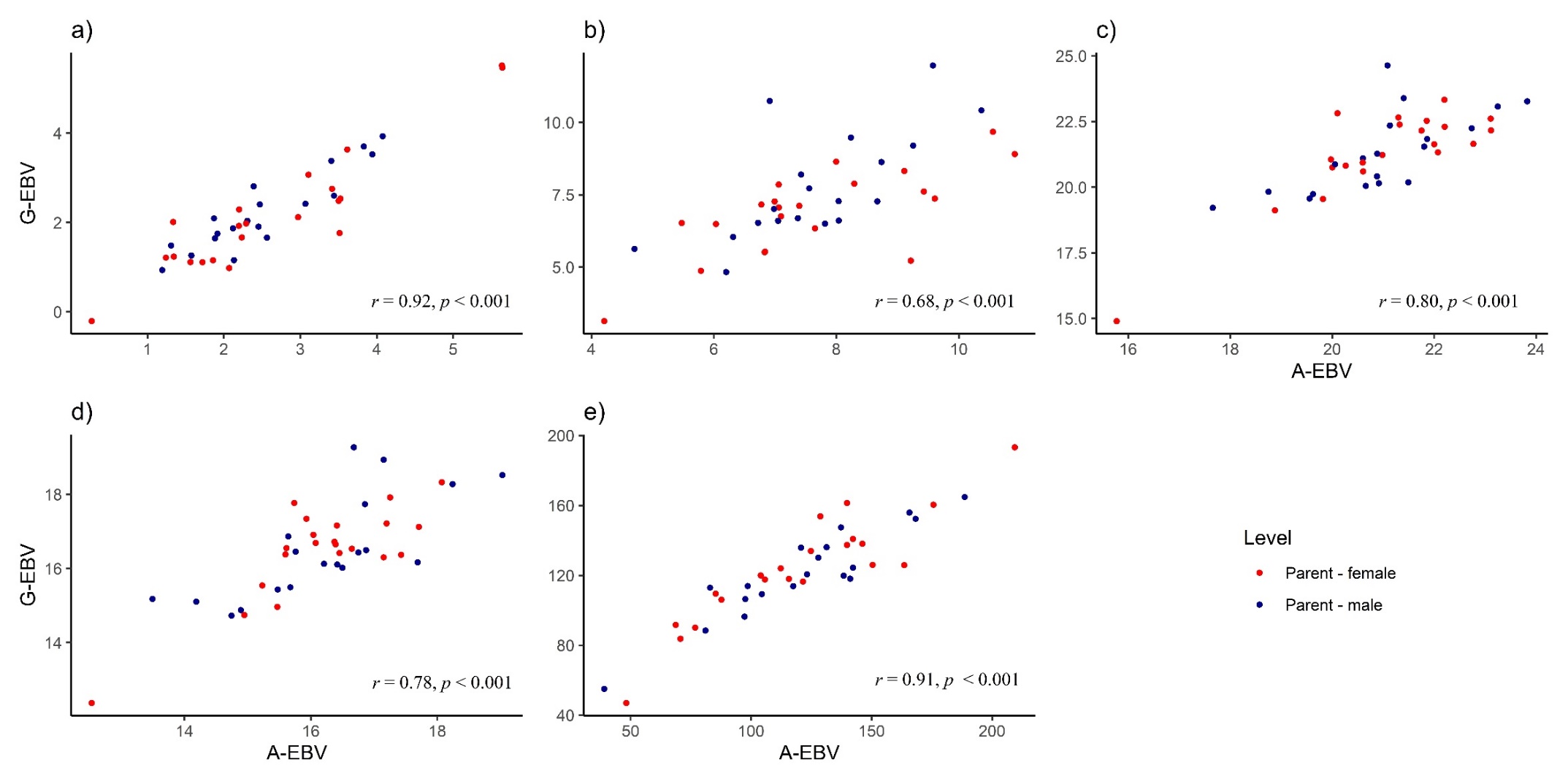


**Supplementary Fig. 2** Parental breeding value correlation. The correlation of pedigree-based (A) and marker-based (G) estimated breeding values (EBV) for parental genotypes using five quantitative traits: **a)** scored fruit load (0.5‒9), **b)** average fruit weight (g), **c)** average dry matter percentage (%), **d)** ripe soluble solids content (°Brix), and **e)** vitamin C content (mg/100 g fresh weight). Parental genotypes were grouped by sex (female = red, male = blue)
