# Supplementary material for: Using genomic selection to correct pedigree errors in kiwiberry breeding
Source: Mol Breed. 2025 Mar 11;45(3):33. doi: 10.1007/s11032-025-01552-6 (PMC11896956; doi:10.1007/s11032-025-01552-6)
Supplement: Supplementary file 2 — Supplementary file2 (DOCX 31.9 KB) [file 11032_2025_1552_MOESM2_ESM.docx]

**Using genomic selection to correct pedigree errors in kiwiberry breeding**

Molecular Breeding

Daniel Mertten*, Catherine M. McKenzie, Susan Thomson, John McCallum, Dave Andersen, Samantha Baldwin, Michael Lenhard, Paul M. Datson

***Corresponding author:**

Daniel Mertten

The New Zealand Institute for Plant and Food Research Limited (PFR)

Auckland 1142, New Zealand

Email: merttendan@gmail.com

**Supplementary Table 1** Number of progeny from a two-factorial breeding scheme, after consider pedigree records. **a)** Two female parents^(common)^ (red) were crossed with 13 male parents^(distinctive)^ (blue). **b)** Thirteen female parents^(distinctive)^ (red) were crossed with three male cultivars^(common)^ (blue). It should be noted that the 13 male parents in **(a)** and the 13 female parents in **(b)** shared the same ancestor within a row, represented in grey. For instance, “AA-D02-02-M” and “AA-D02-01-F” were selections from the same original cross, and consequently, they shared the ancestors (“*Actinidia arguta* D”, “*A. arguta* 02”). The number of progeny indicates successful crosses

| **a)** | |  | | | **b)** | | | |
| --- | --- | --- | --- | --- | --- | --- | --- | --- |
| **Ancestors** | | **Mother** | *A. arguta* B | *A. arguta* A |  | | | |
|  |  | **Father** | *A. arguta* 07 | *A. arguta* 01 |  |  |  |  |
| **Mother** | **Father** | **Male Parent ^(distinctive)^** | **Female Parent ^(common)^** | | **Female Parent ^(distinctive)^** | **Male Parent ^(common)^** | | |
|  |  |  | AA-B07-01-F | AA-A01-03-F |  | *A. arguta* 03 | *A. arguta* 07 | *A. melanandra* 01 |
| *A. arguta* C | *A. arguta* 01 | AA-C01-02-M | 39 | 6 | AA-C01-01-F | 38 | 40 | 27 |
| *A. arguta* C | *A. arguta* 02 | AA-C02-01-M | 39 | 11 | - |  |  |  |
| *A. arguta* C | *A. arguta* 02 | AA-C02-02-M | 35 |  | - |  |  |  |
| *A. arguta* C | *A. arguta* 03 | AA-C03-03-M | 42 | 26 | AA-C03-01-F | 37 | 39 | 16 |
| *A. arguta* C | *A. arguta* 03 | - |  |  | AA-C03-02-F | 36 | 37 | 13 |
| *A. arguta* C | *A. arguta* 04 | AA-C04-02-M | 40 |  | AA-C04-01-F | 39 | 42 | 38 |
| *A. arguta* C | *A. arguta* 05 | AA-C05-02-M | 37 | 32 | AA-C05-01-F | 6 | 19 | 37 |
| *A. arguta* C | *A. arguta* 06 | AA-C06-01-M | 37 |  | - |  |  |  |
| *A. arguta* D | *A. arguta* 01 | - |  |  | AA-D01-01-F | 41 | 38 |  |
| *A. arguta* D | *A. arguta* 01 | - |  |  | AA-D01-02-F | 40 | 30 | 10 |
| *A. arguta* D | *A. arguta* 02 | AA-D02-02-M | 37 | 38 | AA-D02-01-F | 41 | 41 | 32 |
| *A. arguta* D | *A. arguta* 04 | - |  |  | AA-D04-01-F | 36 | 37 | 7 |
| *A. arguta* D | *A. arguta* 05 | AA-D05-01-M | 41 | 39 | - |  |  |  |
| *A. arguta* D | *A. arguta* 06 | AA-D06-02-M | 39 | 2 | AA-D06-01-F | 38 | 38 |  |
| *A. arguta* D | *A. arguta* 07 | AA-D07-02-M | 3 | 27 | AA-D07-01-F | 39 | 20 |  |
| *A. arguta* D | *A. arguta* 07 | AA-D07-04-M | 38 | 39 | AA-D07-03-F | 39 | 40 | 30 |
| *A. arguta* D | *A. arguta* 07 | AA-D07-06-M | 39 |  | AA-D07-05-F | 38 | 38 | 9 |

**Supplementary Table 2** Fruit load score. Fruit numbers in *Actinidia arguta* and hybrids of *A. arguta* × *A. melanandra* converted to score classes are shown

| **Score** | **No. of fruits** |
| --- | --- |
| 0 | 0 |
| 0.5 | 1–4 |
| 1 | 5–10 |
| 2 | 11–30 |
| 3 | 31–60 |
| 4 | 61–100 |
| 5 | 101–200 |
| 6 | 201–300 |
| 7 | 301–400 |
| 8 | 401–500 |
| 9 | ≥ 501 |

**Supplementary Table 3** Properties of the assessed traits: scored fruit load (0.5‒9), fruit weight (g), dry matter (%), ripe soluble solids content (°Brix), and vitamin C (mg/100 g fresh weight). The summary includes the number of observations (N), minimum (Min), first quartile (1^st^ Qu), median, mean, third quartile (3^rd^ Qu), maximum (Max), skewness, years of observation, and results of the Wald Chi-squared test (*χ*² and *p*-value)

| **Trait** | **N** | **Min** | **1^st^ Qu** | **Median** | **Mean** | **3^rd^ Qu** | **Max** | **Skewness** | **No. of years** | **Wald Chi-squared Test** | |
| --- | --- | --- | --- | --- | --- | --- | --- | --- | --- | --- | --- |
|  |  |  |  |  |  |  |  |  |  | ***χ*^2^** | ***p*-value** |
| **Fruit Load** | 838 | 0.50 | 3.0 | 5.0 | 4.4 | 6.0 | 9.0 | 0.1 | 2 | 295.3 | <0.001 |
| **Fruit Weight** | 832 | 1.0 | 6.3 | 7.7 | 7.9 | 9.3 | 17.3 | 0.5 | 3 | 541.7 | <0.001 |
| **Dry Matter** | 825 | 12.0 | 18.5 | 20.6 | 20.6 | 22.5 | 29.3 | 0.1 | 3 | 1163.0 | <0.001 |
| **Ripe Soluble Solids Content** | 809 | 9.1 | 14.3 | 15.9 | 16.0 | 17.6 | 22.4 | 0.2 | 2 | 415.0 | <0.001 |
| **Vitamin C** | 364 | 16.8 | 94.8 | 126.4 | 141.7 | 184.8 | 342.5 | 0.7 | 1 | - | - |

**Supplementary Table 4** Progeny numbers before and after correcting the male parent. The male parent (blue) of four *Actinidia arguta* × *A. melanandra* crosses was corrected from *A. melanandra* 01 to *A. arguta* 07 using genetic marker information. The female parents (red) remained unaltered. The original number of progeny is shown in brackets

| **Female Parent** | **Male Parent** | |
| --- | --- | --- |
|  | *A. arguta* 07 | *A. melanandra* 01 |
| AA-C03-01-F | 55 \| (39) | 0 \| (16) |
| AA-C04-01-F | 80 \| (42) | 0 \| (38) |
| AA-C05-01-F | 46 \| (19) | 10 \| (37) |
| AA-D01-02-F | 40 \| (30) | 0 \| (10) |

**Supplementary Table 5** Breeding value correlations of five fruit traits were assessed across various sources of information. The Pearson correlation of pedigree- and marker-based estimated breeding values was analysed for scored fruit load (0.5‒9), fruit weight (g), dry matter (%), ripe soluble solids content (°Brix), and vitamin C (mg/100 g fresh weight). Estimated breeding values, based on marker-derived relationship matrix, were compared with pedigree-based breeding values derived from unaltered pedigrees (*A*^true^), pedigrees with randomised common (*A*^common^) and distinctive (*A*^distinctive^) parents, as well as combinations thereof at both the cross level (*A*^random^) and individual progeny level (*A*^random*^). Correlations and corresponding *p*-values are presented for the parental generation, female progeny (validation set), and male progeny lacking observational records. Additionally, correlations of female progeny (training set) with available observations are included

| **Trait** | **Breeding value correlation – parental** | | | | | | | | | |
| --- | --- | --- | --- | --- | --- | --- | --- | --- | --- | --- |
|  | ***G*~*A*^true^** | ***p*-value** | ***G*~*A*^common^** | ***p*-value** | ***G*~*A*^distinctive^** | ***p*-value** | ***G*~*A*^random^** | ***p*-value** | ***G*~*A*^random*^** | ***p*-value** |
| **Fruit Load** | 0.92 | <0.001 | 0.54 | <0.001 | 0.34 | 0.034 | 0.01 | 0.940 | 0.08 | 0.610 |
| **Fruit Weight** | 0.68 | <0.001 | 0.56 | <0.001 | 0.41 | <0.010 | 0.34 | 0.031 | 0.48 | <0.010 |
| **Dry Matter** | 0.80 | <0.001 | 0.36 | 0.023 | 0.56 | <0.001 | 0.20 | 0.230 | 0.18 | 0.270 |
| **Ripe Soluble Solids Content** | 0.78 | <0.001 | 0.26 | 0.110 | 0.59 | <0.001 | 0.19 | 0.250 | 0.20 | 0.220 |
| **Vitamin C** | 0.91 | <0.001 | 0.63 | <0.001 | 0.25 | 0.120 | 0.04 | 0.830 | 0.13 | 0.430 |
|  | **Breeding value correlation – female progeny ^(training)^** | | | | | | | | | |
| **Fruit Load** | 0.96 | <0.001 | 0.89 | <0.001 | 0.94 | <0.001 | 0.90 | <0.001 | 0.86 | <0.001 |
| **Fruit Weight** | 0.98 | <0.001 | 0.97 | <0.001 | 0.98 | <0.001 | 0.98 | <0.001 | 0.97 | <0.001 |
| **Dry Matter** | 0.95 | <0.001 | 0.91 | <0.001 | 0.93 | <0.001 | 0.91 | <0.001 | 0.89 | <0.001 |
| **Ripe Soluble Solids Content** | 0.94 | <0.001 | 0.86 | <0.001 | 0.91 | <0.001 | 0.88 | <0.001 | 0.83 | <0.001 |
| **Vitamin C** | 0.89 | <0.001 | 0.70 | <0.001 | 0.81 | <0.001 | 0.73 | <0.001 | 0.33 | <0.001 |
|  | **Breeding value correlation – female progeny ^(validation)^** | | | | | | | | | |
| **Fruit Load** | 0.90 | <0.001 | 0.42 | <0.001 | 0.86 | <0.001 | 0.61 | <0.001 | 0.01 | 0.770 |
| **Fruit Weight** | 0.72 | <0.001 | 0.70 | <0.001 | 0.69 | <0.001 | 0.67 | <0.001 | 0.57 | <0.001 |
| **Dry Matter** | 0.74 | <0.001 | 0.51 | <0.001 | 0.62 | <0.001 | 0.52 | <0.001 | 0.03 | 0.450 |
| **Ripe Soluble Solids Content** | 0.81 | <0.001 | 0.48 | <0.001 | 0.70 | <0.001 | 0.58 | <0.001 | 0.07 | 0.035 |
| **Vitamin C** | 0.91 | <0.001 | 0.58 | <0.001 | 0.85 | <0.001 | 0.70 | <0.001 | 0.16 | <0.010 |
|  | **Breeding value correlation – male progeny** | | | | | | | | | |
| **Fruit Load** | 0.90 | <0.001 | 0.44 | <0.001 | 0.85 | <0.001 | 0.61 | <0.001 | 0.01 | 0.710 |
| **Fruit Weight** | 0.74 | <0.001 | 0.71 | <0.001 | 0.70 | <0.001 | 0.68 | <0.001 | 0.60 | <0.001 |
| **Dry Matter** | 0.78 | <0.001 | 0.52 | <0.001 | 0.66 | <0.001 | 0.53 | <0.001 | 0.06 | 0.059 |
| **Ripe Soluble Solids Content** | 0.83 | <0.001 | 0.50 | <0.001 | 0.72 | <0.001 | 0.58 | <0.001 | 0.07 | 0.029 |
| **Vitamin C** | 0.89 | <0.001 | 0.43 | <0.001 | 0.79 | <0.001 | 0.56 | <0.001 | 0.07 | 0.026 |
